# *Vincetoxicum arnottianum* modulates motility features and metastatic marker expression in pediatric rhabdomyosarcoma by stabilizing the actin cytoskeleton

**DOI:** 10.1186/s12906-021-03299-x

**Published:** 2021-05-04

**Authors:** Anna Adamus, Iftikhar Ali, Vasileios Vasileiadis, Luai Al-Hileh, Jan Lisec, Marcus Frank, Guido Seitz, Nadja Engel

**Affiliations:** 1 Department of Pediatric Surgery, University Hospital, Marburg, Germany; 2 Department of Chemistry, Karakoram International University, Gilgit, Pakistan; 3 Shandong Key Laboratory of TCM Quality Control Technology, Shandong Analysis and Test Center, Jinan, Shandong Province P.R. China; 4 Division 1.7 Analytical Chemistry, Federal Institute for Materials Research and Testing (BAM), Berlin, Germany; 5 Medical Biology and Electron Microscopy Center, Rostock University Medical Center, Rostock, Germany; 6 Department of Life, Light & Matter, University of Rostock, Rostock, Germany; 7 Department of Oral and Maxillofacial Surgery, Facial Plastic Surgery, Rostock University Medical Center, Rostock, Germany

**Keywords:** Alveolar rhabdomyosarcoma, Vincetoxicum, Metastasis, Migration, Invasion

## Abstract

**Background:**

Prevention of metastatic invasion is one of the main challenges in the treatment of alveolar rhabdomyosarcoma. Still the therapeutic options are limited. Therefore, an anti-tumor screening was initiated focusing on the anti-metastatic and anti-invasion properties of selected medicinal plant extracts and phytoestrogens, already known to be effective in the prevention and treatment of different cancer entities.

**Methods:**

Treatment effects were first evaluated by cell viability, migration, invasion, and colony forming assays on the alveolar rhabdomyosarcoma cell line RH-30 in comparison with healthy primary cells.

**Results:**

Initial anti-tumor screenings of all substances analyzed in this study, identified the plant extract of *Vincetoxicum arnottianum* (VSM) as the most promising candidate, harboring the highest anti-metastatic potential. Those significant anti-motility properties were proven by a reduced ability for migration (60%), invasion (99%) and colony formation (61%) under 48 h exposure to 25 μg/ml VSM. The restricted motility features were due to an induction of the stabilization of the cytoskeleton – actin fibers were 2.5-fold longer and were spanning the entire cell. Decreased proliferation (PCNA, AMT, GCSH) and altered metastasis (e. g. SGPL1, CXCR4, stathmin) marker expression on transcript and protein level confirmed the significant lowered tumorigenicity under VSM treatment. Finally, significant alterations in the cell metabolism were detected for 25 metabolites, with levels of uracil, N-acetyl serine and propanoyl phosphate harboring the greatest alterations. Compared to the conventional therapy with cisplatin, VSM treated cells demonstrated a similar metabolic shutdown of the primary cell metabolism. Primary control cells were not affected by the VSM treatment.

**Conclusions:**

This study revealed the VSM root extract as a potential, new migrastatic drug candidate for the putative treatment of pediatric alveolar rhabdomyosarcoma with actin filament stabilizing properties and accompanied by a marginal effect on the vitality of primary cells.

**Graphical abstract:**

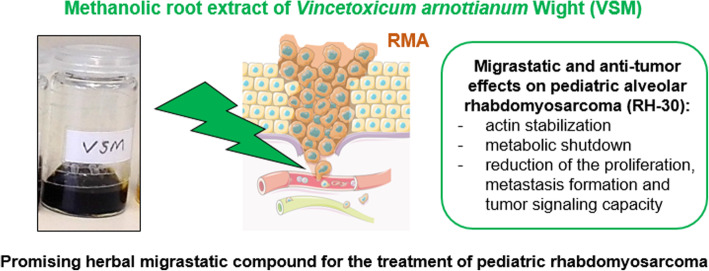

**Supplementary Information:**

The online version contains supplementary material available at 10.1186/s12906-021-03299-x.

## Background

Rhabdomyosarcoma (RMS) is the most common pediatric soft tissue tumor and accounts for about 6–8% of all pediatric malignancies [1]. The RMS tumor type is derived from skeletal muscle cells and arises due to a failure in the myogenic differentiation program [2, 3]. RMS can be histologically classified in several subtypes. The two most common subtypes are the embryonal (RME; 70%) and the alveolar (RMA; 20%) rhabdomyosarcomas [4, 5]. The most frequent cause of treatment failure is local failure. Another crucial issue in treating RMS-patients is preventing and treating metastasis. As in most cancer cases, these patients have a much poorer prognosis than those without metastatic events [6–9]. RMA shows frequent molecular alterations, which are associated with a poorer prognosis [4, 10, 11]. For example, gene fusions lead to a subclassification in translocation positive (80%) and translocation negative (20%) subtypes. The translocation positive subtypes are characterized by the expression of pax3/7-foxo1 fusion transcription factors which promote pathogenesis, oncogenesis and the formation of metastasis as well as drug resistance mechanism in these cancer types [2, 10–13]. The established multimodal treatment approaches consist of a polychemotherapy combined with local control strategies using surgery and/or radiotherapy [1, 14]. However, established therapies often fail in advanced RMA and tumor relapse cases, rendering novel treatment strategies necessary [1, 2].

In order to reduce the mortality and morbidity rates in cancer patients, many studies aim to understand metastasis occurrence and develop prevention strategies, since most of the cancer patients succumb to metastases and not to the primary tumor [15–17]. A promising new drugs generation are migrastatics, which intend to avoid metastatic invasion by targeting the cytoskeleton with synthetic and nature-derived components. The primary target of these drugs is the stabilization of the actin cytoskeleton. Furthermore, the acto−/ tropo−/ myosin contractility or the ion and energy sources required during migration can be targeted by migrastatics [18–20]. By directly affecting the cytoskeleton, the motility properties of the cells are also modulated, thus developing new therapeutics against metastasis events.

Our previous in vitro studies successfully evaluated the anti-adhesive and actin cytoskeleton-stabilizing effects of selected medicinal plant extracts from Pakistan and Europe in breast cancer and osteosarcoma [21, 22]. In this context the effect of two selected plant-derived extracts of the medicinal plant species i.e., *Vincetoxicum arnottianum* (VSM; methanol whole plant extract) and *Linum usitatissimum L.* (LW; ethanol root extract) were evaluated. The main effective ingredients of Linum were already analyzed by mass spectrometry in one of our former projects [22]. We primarily identified isoflavones and lignans, for example secoisolariciresinol, matairesinol, biochanin, daidzein and glycites - classical phytoestrogens, whose anti-cancer effects have already been proven. Therefore, selected single phytoestrogens, abbreviated as secoisolariciresinol (Seco), matairesinol (Mata), daidzein (Daid) and genistein (Geni) were evaluated on the metastasizing assets of alveolar RMS cells. The secondary plant compounds Seco, Mata, Daid are known to reduce tumor growth of breast and prostate cancer cells [22–26]. The secondary plant substance Geni is a main ingredient of soy (*Glycine max L.*), and several experimental and clinical investigations suggest a therapeutic role of Geni on different types of cancer [27, 28].

Our primary aim was to identify potential plant-derived substances which can reduce the RMA migration, invasion, and anchorage-independent colony formation capacity in vitro. The suitable candidates were then examined in more detail to clarify the underlying mechanisms of action, by three different microscopy techniques, RT-PCR and western blot analysis as well as metabolic profiling.

## Methods

### Chemicals

The isoflavones genistein (4′,5,7-Trihydroxyisoflavone; abbreviated as ‘Geni’) and daidzein (4′,7-Dihydroxyisoflavone 7-glucoside; abbreviated as ´Daid´), the lignans matairesinol ((αR,βR)-α,β-Bis (4-hydroxy-3-methoxybenzyl) butyrolactone; abbreviated as ´Mata´) and secoisolariciresinol ((2R,3R)-2,3-Bis (4-hydroxy-3-methoxybenzyl)-1,4-butanediol; abbreviated as ´Seco´) as well as the actin-specific reagent and positive control jasplakinolide (jaspamide; abbreviated as ´Jaspl.´) were purchased from Sigma Aldrich (Steinheim, Germany). All the plant-derived single compounds were stored at − 20 °C in the dark as single-used aliquots of concentrated stock solutions in dimethylsulfoxide (DMSO). The anti-cancer chemotherapy drug cisplatin (Chem Cruz, Dallas, USA) was used as positive control in the metabolic profiling studies. Its stock solution was prepared according to the equitoxic formulation in humans (3.3 mM in 0.9% saline with 10 mg/ml mannitol).

### Plant material collection and identification

The *Vincetoxicum arnottianum* Wight (VSM) plant extract was provided by Dr. Iftikhar Ali (Karakoram International University, Department of Chemistry, Gilgit, Pakistan) and its collection and identification were described previously [21]. Briefly, it was collected from Baluchistan (Pakistan) and its authentication was carried out by the plant taxonomists Prof. Rasool Bakhsh Tareen (Department of Botany, University of Balochistan, Quetta, Pakistan) and Dr. Sher Wali Khan (Department of Biological Sciences, Karakoram International University, Gilgit, Pakistan). The VSM plant sample was extracted in MeOH and 50 mg of its dry sample was dissolved in 1 ml DMSO to a 50 mg/ml stock solution. The *Linum usitatissimum* (LW) plant material and extract preparation from native flax roots was described previously [22]. Briefly, the seeds were obtained from the Agriculture Research Institution (LUFA, Rostock, Germany) and the plants were raised in the University of Rostock (Rostock, Germany). The dry extract powder was dissolved in EtOH to a stock solution of 100 mg/ml. The stock solutions of plant-derived extracts were further diluted in different concentrations for the biological investigation and anti-tumor activity testing. The main secondary plant compounds of *Vincetoxicum arnottianum* (VSM) and *Linum usitatissimum* L. (LW) were previously identified by NMR and LC-MS analysis in accordance to their retention time [22, 29] and listed in Table 1.

**Table 1:** Main secondary plant compounds of *Vincetoxicum arnottianum* (VSM) and *Linum usitatissimum* L. by NMR and LC-MS analysis in accordance to their retention time [22, 29]

*Vincetoxicum arnottianum* (VSM)	*Linum usitatissimum* L. (LW)
ß-Sitosterol	Secoisolariciresinol
β-sitosterol-β-D-glucoside	Lariciresinol
Lupeol	Matairesinol
	Pinoresinol
	Arctigenin
	Biochanin
	Fisetin
	Daidzein
	Glyciten

### Cell culture and extract treatment procedure

The RMA cell line RH-30 (ACC-489) was obtained from the German biological resource bank ‘DSMZ’ (https://www.dsmz.de), HA-OH was a gift from Prof. Dr. E. Koscielniak (Olgahospital, Klinikum Stuttgart), RD (ATCC® CCL-136™) and A204 were purchased from ATCC (ATCC® HTB82™). All cell lines were cultured in Dulbecco’s modified Eagle’s medium plus Ultraglutamine 1 (Lonza, Verviers, Belgium), with 10% fetal calf serum (PAN Biotech GmbH, Aidenbach, Germany) and 1% Antibiotic-Antimycotic-Solution (Gibco, Paisley, UK) and maintained at 37 °C and in a 5% CO_2_ atmosphere. Every second day the culture medium was changed, and confluent cancer cells were treated with 0.05% trypsin – 0.02% EDTA (Lonza, Verviers, Belgium). Under assay conditions, the fetal calf serum was replaced by a charcoal stripped fetal calf serum (Pan-Biotech GmbH, Aidenbach, Germany) and the Dulbecco’s modified Eagle’s medium was replaced by a phenol-red-free Dulbecco’s modified Eagle’s medium (PAA Laboratories GmbH, Germany) to avoid unspecific stimulations of the culture medium. Prior treatment, the cells were adapted for 48 h to the assay medium. The plant extract and plant-derived compounds were applied to the assay medium in different concentrations for 48 h. The vehicle 0.1% DMSO (Sigma-Aldrich, Steinheim, Germany) functions as negative control. As primary non-tumorigenic control cells human fibroblasts (NHDF, C-12385) and mesenchymal stem cells (hMSC, C-12977) were purchased from PromoCell GmbH, Heidelberg, Germany and cultivated in the appropriate medium that Promocell also offers.

### Cell viability assay

The cell viability of the cells after 48 h treatment with different concentrations of plant extracts (VSM and LW) and plant-derived compounds (Geni, Seco, Mata, Daid and Jaspl.) compared to the treatment with the vehicle control (0.1% DMSO) was quantified with the CellTiter 96®AQueous One Solution Cell Proliferation Assay Kit (MTS) (Promega Corp., Madison, USA) according to the manufacturer’s instruction manuals as described previously [21, 30]. The photometric absorption was read at λ = 490 nm against a reference at λ = 600 nm, using MRX Revelation 4.06 microplate reader (Dynex Technologies, USA) and at least, eight replicates with corrected background absorbance were conducted.

### Cell migration assay

The analysis of cell migration capacity under treatment with different concentrations of plant extracts (VSM and LW) and plant-derived compounds (Geni, Seco, Mata, Daid and Jaspl.) was performed according to the Ibidi protocol with Ibidi culture inserts (μ-Dish 35 mm; Ibidi GmbH, Martinsried, Germany) and gap closure was analyzed as described previously [21, 30, 31]. The images during gap closure were taken with the bright field microscope (CKX53, Olympus, Tokyo, Japan) and the gap area [μm^2^] was evaluated with the software CellSens Entry (Olympus, Tokyo, Japan).

### Cell invasion assay

Cell invasion assay was performed as described previously [30, 31], with the 12-well tissue culture (TC) inserts (Ø 8.0 μm pore size) (Sarstedt, Germany), which were coated over night with 10 μg/ml MaxGel™ ECM (Sigma Aldrich, Saint Luis, USA). Cells were 48 h adapted in assay medium, 48 h pre-treated with different concentrations of plant extract (VSM and LW) and plant-derived compounds (Geni, Seco, Mata, Daid and Jaspl.), and seeded in a density of 15 × 10^3^ cells per milliliter in the upper chamber of the membrane insert. Afterwards, the cells were 24 h exposed to a concentration gradient (upper chamber: DMEM without phenol red and with 10% FCS; lower chamber: DMEM with 20% FCS). The invaded cells were fixed and stained for 30 min with 0.5% crystal violet (Sigma Aldrich, Saint Luis, USA) and 6% glutaraldehyde (Santa Cruz, Dallas, USA) in PBS. The images of invaded cells were taken the bright field microscope (CKX53, Olympus, Tokyo, Japan) and cells were counted with the software CellSens Entry (Olympus, Tokyo, Japan).

### Colony forming assay

The analysis of the capacity for colony formation in soft agar during treatment was conducted according to the protocol of Borowicz et al. [32] and Engel et al. [22]. Briefly, cells were 48 h adapted in assay medium and further treated for 48 h with different concentrations of plant extract (VSM and LW) and plant-derived compounds (Geni, Seco, Mata, Daid and Jaspl.). The bottom layer in each well of a 6-well plate (Sarstedt, Germany) was made with 1.5 ml of 1% agar (Sigma Aldrich, Saint Luis, USA) in assay medium and the top layer was made with 1.5 ml of 0.6% agar and 5000 cells in assay medium (i.e., 0.75 ml assay medium with 5000 cells and 0.75 ml 0.6% agarose solution). After 3–4 weeks of growth the cell colonies were fixed and stained with 6% glutaraldehyde (Santa Cruz, Dallas, USA) and 0.5% crystal violet (Sigma Aldrich, Saint Luis, USA) in PBS. The images of colonies were taken with the bright field microscope (CKX53, Olympus, Tokyo, Japan). Colonies containing more than 25 cells were counted.

### Bright field microscopical visualization of cell morphology

The alterations in the cellular morphology under treatment with VSM concentration series (100, 50, 25, 10 and 1 μg/ml) were analyzed with a CXK53 inverse bright field microscope (Olympus, Tokyo, Japan). The images were taken via the software CellSens Entry (Olympus, Tokyo, Japan). For the quantification of morphological changes, especially vesicle formation, which occurred under VSM treatment (Figs. 3a – b) the images were divided into several quadrants and the total number of vesicles per image was counted using the microscopy software Cell Sens Entry (Olympus, Japan). At least 5 images per treatment approach were included to the analysis.

### Actin visualization by fluorescence microscopy and quantification

For fluorescence labelling of 48 h VSM (25 μg/ml) treated cells, the cells were grown in Ibidi dishes (Ibidi GmbH, Martinsried, Germany), fixed in 4% paraformaldehyde (Santa Cruz, Dallas, USA), permeabilized with 0.1% Triton X-100 (Santa Cruz, Dallas, USA) and labelled with F-Actin antibody Phalloidin-Alexa 596 (Invitrogen, USA). Afterwards, a counter-staining with Hoechst (PanReacAppliChem, Darmstadt, Germany) was performed. The labelling procedure was also described previously [21, 30]. The fluorescence microscopical images were captured on a confocal laser-scanning microscope Leica DMi8 (Leica, Wetzlar, Germany). For subsequent quantification of F-actin fibers (stress fibers induction), the confocal images were mathematical processed with the software FilaQuant (University of Rostock, Institute of Mathematics, Mathematical Optimization, Rostock, Germany) as described previously [22, 33].

### SEM visualization of cell morphology alterations

For scanning electron microscopy (SEM) cells were grown on glass cover slips (Menzel-Gläser GmbH, Darmstadt, Germany) and incubated for 48 h with 25 μg/ml VSM under treatment conditions. The cells were fixed with 2% glutaraldehyde and 1% paraformaldehyde, washed in 0.1 M phosphate buffer and dehydrated with a graded series of ethanol and finally processed for critical point drying using CO_2_ as intermedium (Emitech K850 critical point dryer, Emitech Ltd. Ashford, UK) as described previously [30]. The cover slips were mounted on SEM stubs with adhesive carbon tape (Plano, Wetzlar, Germany) and sputter-coated with a gold layer (approximately 15–20 nm thickness) using a Bal-Tec SCD004 sputter coater (Balzers Union Ltd., Balzers, Liechtenstein). Specimens were viewed in a field-emission SEM operated at 5 kV (Merlin VP compact, Carl Zeiss Microscopy, Jena, Germany) and images with a size of 1024 × 768 pixels were recorded.

### Transcript expression analysis

The RNA isolation was done using the Aurum™ Total RNA Mini Kit from Bio-Rad (USA) and cDNA synthesis was performed using RevertAid First Strand cDNA Synthesis Kit (#K1622) (Thermo Fisher Scientific Inc., Rockford, IL, USA), both according to the product protocol. RT-PCR was performed as described previously [31, 34] using the primer pairs listed in Table 2 and Dream Taq™ Green PCR Master Mix (Thermo Fisher Scientific Inc., Vilnius, Lithuania) in the Eppendorf Mastercycler® ‘Mastercycler gradient’ (Eppendorf AG, Hamburg, Germany).

**Table 2:** Overview and sequence of all used primer pairs for transcript amplification with RT-PCR

Name	Forward Primer	Reverse Primer
**β-Actin**	5′-GGGCATGGGTCAGAAGGATT-3′	5′-GAGGCGTACAGGGATAGCAC-3′
**GAPDH**	5′-CAAGGTCATCCATGACAACTTTG-3′	5′-GTCCACCACCCTGTTGCTGTAG-3′
**PAX3-FOXO1**	5′-GCACTGTACACCAAAGCACG-3′	5′-CTGTGGATTGAGCATCCACC-3′
**GLDC**	5′-GTTCCAGTACCCAGACACGG-3′	5′-GCACTCCTCTCCCATGCTTT-3′
**AMT**	5′-CAGTACCGGGACAGTCACAC-3′	5′-ACAGCACTGGTCATGAAGGG-3′
**GCSH**	5′-GTCTCCCTGAAGTTGGGACA-3‘	5′-TCTGAAGGGTTACTCAGTGTCA-3‘
**DLD**	5′-ATGCTGGCTCACAAAGCAGA-3‘	5′-CCAGCACCTGGTCCAAGAAT-3‘
**SGPL1**	5′-ACTGCTCGCTTCCTCAAGTC-3‘	5′-GTGACAGTGTCGGTGCTGTA-3‘
**SPHK1**	5′-TGGCGTCATGCATCTGTTCT-3‘	5′-AGTAGTTTGGGTGCACCTGG-3‘
**SPHK2**	5′-TCGTTCTGTGTCTGACCTGC-3‘	5′-CATGAGCACAAAGTCCCCCT-3‘
**Ezrin**	5′-TGCGGAGCTTGCAGAATACA-3‘	5′-GGATGCCCTCACTAGACAGC-3‘
**CXCR4**	5′-TCCATTCCTTTGCCTCTTTTGC-3‘	5′-CCAGACGCCAACATAGACCA-3‘
**P-Cadherin**	5′-ACGACGGGGACCATTTTACC-3‘	5′-ACCTCTGCCGTCCAGTAGAT-3‘
**Syndecan-4**	5′-GACGATGAGGATGTAGTGGG-3‘	5′-CCAGGTCATAGCTGCCTTCA-3‘
**Prominin**	5′-AGAAATGCACCAGCGACAGA-3‘	5′-ACGCCTTGTCCTTGGTAGTG-3‘
**β-Catenin**	5′-GCTTTCAGTTGAGCTGACCA-3‘	5′-CAAGTCCAAGATCAGCAGTCTC-3‘

### Western blotting

Western blot analysis was performed as already described [21, 30, 34]. Briefly, for protein detection, primary antibodies anti-β-Actin ((C4) #sc-47,778; Santa Cruz, Dallas, USA), anti-PCNA ((PC10) #sc-56; Santa Cruz, Dallas, USA), anti-AMT (#10633–1-AP; Proteintech Europe, Manchester, UK), anti-GCSH (#16726–1-AP; Proteintech Europe, Manchester, UK), anti-SGPL1 ((H-300) #sc-67,368; Santa Cruz, Dallas, USA), anti-Ezrin ((3C12) #sc-58,758; Santa Cruz, Dallas, USA), anti-CXCR4 (#11073–2-AP; Proteintech Europe, Manchester, UK) P-Cadherin (#13773–1-AP; Proteintech Europe, Manchester, UK) and Stathmin (#3352; Cell Signaling, Danvers, USA) were incubated overnight at 4 °C followed by labelling with a horseradish peroxidase (HPR)-conjugated secondary antibody (mouse #7076; rabbit #7074P2; Cell Signaling, Danvers, USA) for 1 h at room temperature. Finally, the protein signals were visualized with the Clarity™ Western ECL Chemiluminescent Substrate (Bio-Rad Laboratories Inc., USA). Stain free-images and β-actin were used as loading control. Band intensity was analyzed densitometrically with the Molecular Imager ChemiDoc XRS and Image Lab 6.0.1 software (Bio-Rad, München, Germany). Protein detection was repeated at least three times with individually prepared cell lysates from independent passaged cells.

### Metabolic profiling

The metabolite profiling of 48 h VSM (25 μg/ml) as well as cisplatin (3.3 μM) treated RH-30 cells was conducted by gas chromatography–mass spectrometry (GC–MS) as described previously [30]. For each sample, 600,000 RH-30 cells were harvested with 0.05% trypsin – 0.02% EDTA (Lonza, Maryland, USA), washed three times with ice-cold 0.9% sodium chloride and centrifuged with 14,000 rpm for 2 min at 4 °C. Afterwards, the cell pellet was frozen in liquid nitrogen. The extraction and derivatization procedure were already described by Lisec et al. [35]. 300 μl of cell extract were used for global metabolite profiling analysis. Derivatization and analyses of metabolites were carried out by a GC (7890 A, Agilent, Santa Clara, USA) coupled to a Quadrupol-Time-Of-Flight mass spectrometer (impact II, Bruker Daltonik, Bremen, Germany) via an atmospheric pressure chemical ionization ion source (APCI). Metabolites were identified in comparison to the Golm Metabolome Database [36] or putatively annotated as described previously [37].

### Statistical analysis

Western blotting, RT-PCR’s and Immunofluorescence experiments were replicated at least three times with individually passaged cells, and data sets were expressed as means ± standard deviations (SD). Statistically significant differences were compared using the unpaired Student’s t-test. *P* values: *** *P* < 0.001; ** *P* < 0.01; * *P* < 0.05 were considered statistically as significant. All analyses were performed with the software Microsoft Excel 2017 and Graphpad Prism Version 5 (http://www.graphpad.com/scientific-software/prism/). All statistical tests on metabolite profiles have been conducted in R (www.r-project.org) using the respective functions. For principal component analysis we used the pcaMethods package, applying the nipals algorithm on pareto normalized data. For analysis of variance (ANOVA) we considered genotype and treatment as factors and allowing for interactions. The resulting *P*-values were ranked and corrected according to the original FDR method of Benjamini-Hochberg (Desired FDR (Q) = 5%) with GraphPad Prism 8 (www.graphpad.com).

## Results

### Initial screening on cell viability, migration, invasion, and colony formation

**Fig. 1:**
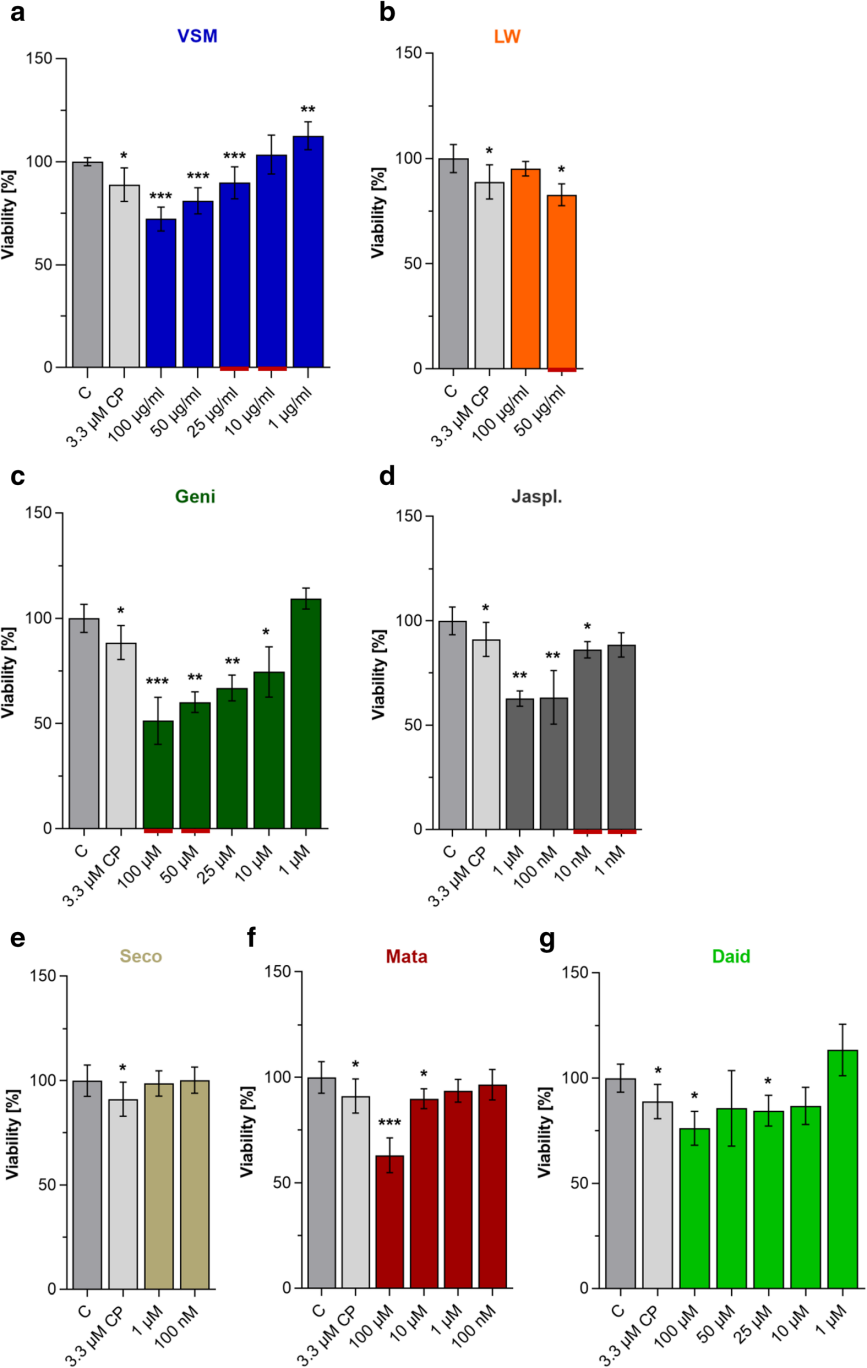
Anti-tumor screening of two plant extracts and five plant derived compounds on the RMA cell line RH-30. **a–g** Cell viability measurement via MTS-assay of RH-30 cells after treatment with two plant extracts (VSM and LW) and five plant-derived compounds (Geni, Seco, Mata, Daid and Jaspl.) at different concentrations for 48 h. The solvent 0.1% DMSO was used as negative control and set to 100%. Mean ± SD, *n* = 6–8, *** *P* < 0.001, ** *P* < 0.01, * *P* < 0.05, significantly different compared to the control, unpaired t-test. The red underlined concentrations of VSM, Geni, LW and Jaspl. were selected for further testing

**Fig. 2:**
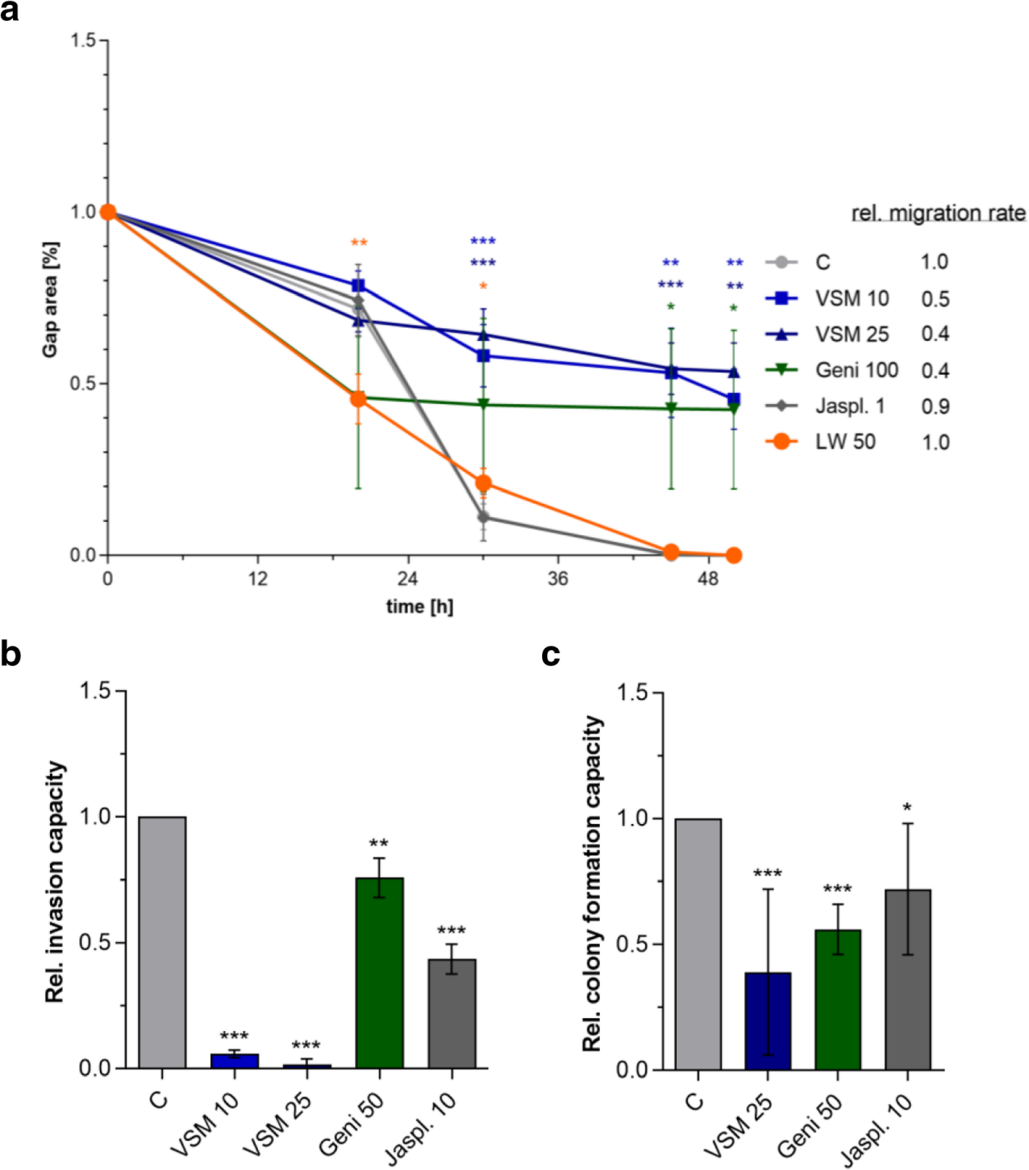
Screening of selected compounds for migrastatic effects on RH-30 cells. **a** Evaluation of cell migration capacity and relative migration speed (ratio) of RH-30 cells during VSM (10 and 25 μg/ml), LW (50 μg/ml), Geni (100 μM) and Jaspl. (1 nM) treatment for 2 days compared to the control (0.1% DMSO). Mean ± SD, *n* = 6–8, *** *P* < 0.001, ** *P* < 0.01, * *P* < 0.05, significantly different compared to vehicle control, unpaired t-test. **b** Determination of the relative invasion capacity of RH-30 cells after 48 h VSM (10 and 25 μg/ml), Geni (50 μM) and Jaspl. (10 nM) treatment compared to the vehicle control (0.1% DMSO). Mean ± SD, *n* = 3, *** *P* < 0.001, ** *P* < 0.01, * *P* < 0.05, significantly different compared to vehicle control, unpaired t-test. **c** Determination of the relative colony formation capacity of RH-30 cells after 48 h VSM (25 μg/ml), Geni (50 μM) and Jaspl. (10 nM) treatment compared to the vehicle control (0.1% DMSO). Mean ± SD, *n* = 3, *** *P* < 0.001, ** *P* < 0.01, * *P* < 0.05, significantly different compared to vehicle control, unpaired t-test. **a-c** Note the individual adjustments in the treatment concentrations of Jaspl. and Geni used within Figs. 2a, b and c are to optimize the respective assay conditions to capture respective minimum effective initial concentration in each graph and to ensure assay function, respectively

To examine the anti-tumor properties of phytoestrogens on pediatric RMA, two promising plant extracts (from Pakistan: VSM and from Germany: LW [21, 22];) and four single phytoestrogens (isoflavones: Geni and Daid; lignans: Seco and Mata) as well as the positive control jasplakinolide, an actin-stabilizing compound (jaspamide: Jaspl.) were selected. Their anti-cancer potential was screened on the human and established RMA cell line RH-30 by cell viability assays (Fig. 1) and subsequently examined with assays to analyze the ability for cell migration, invasion and anchorage-independent colony formation (Fig. 2).

For the initial screening RH-30 cells were treated for 48 h with a concentration series of VSM (100, 50, 25, 10 and 1 μg/ml), Geni and Daid (100, 50, 25, 10 and 1 μM), as well as with different concentrations based on literature data of LW (100 and 50 μg/ml), Jaspl. (1000, 100, 10, 1 nM), Seco (1 and 0.1 μM) and Mata (100, 10, 1 and 0.1 μM) (Figs. 1a-g). VSM extract treatment induced a significant decreased viability rate in a range of 10–30%, respectively (Fig. 1a). The treatment with 50 μg/ml LW extract induced a significant viability decrease of 20% (Fig. 1b). Moreover, treatment with the isoflavone Geni induced a significant decreased viability rate in a range of 25–50%, respectively (Fig. 1c). The highest concentration of the positive control Jaspl. (1 μM) induced a viability decrease of 40% (Fig. 1d). In contrast, Seco treatment exerts no impact on cell viability (Fig. 1e), whereas the treatment with high concentrations (100 μM) of Mata and Daid revealed a significant viability decrease of 40% (Fig. 1f) and 25% (Fig. 1g), respectively.

Based on the screening results, the VSM and the LW extracts, the single compounds Geni and the Jaspl., and the control were used for continuing cell motility examinations (Fig. 2a). Geni100 and the VSM25 exhibit the most promising anti-migratory effects (50–60%, quantitatively determined by the relative migration rate). Both agents were further tested for their effect on RH-30 cell invasion capacity (Fig. 2b). The positive control (10 nM Jaspl.) mediated a 60% reduction and VSM10 and VSM25 showed a 95 and 99% invasion reduction, respectively. Geni50 treatment decreased the invasiveness about 25%. The capacity for anchorage-independent growth and colony formation under VSM25 and Geni50 treatment was also assayed compared to the negative and positive controls (Fig. 2c). The positive control (10 nM Jaspl.) reduced the colony formation about 27%, whereas Geni50 and VSM25 revealed a reduction about 44 and 61%, respectively.

### Morphological alterations under VSM treatment

**Fig. 3:**
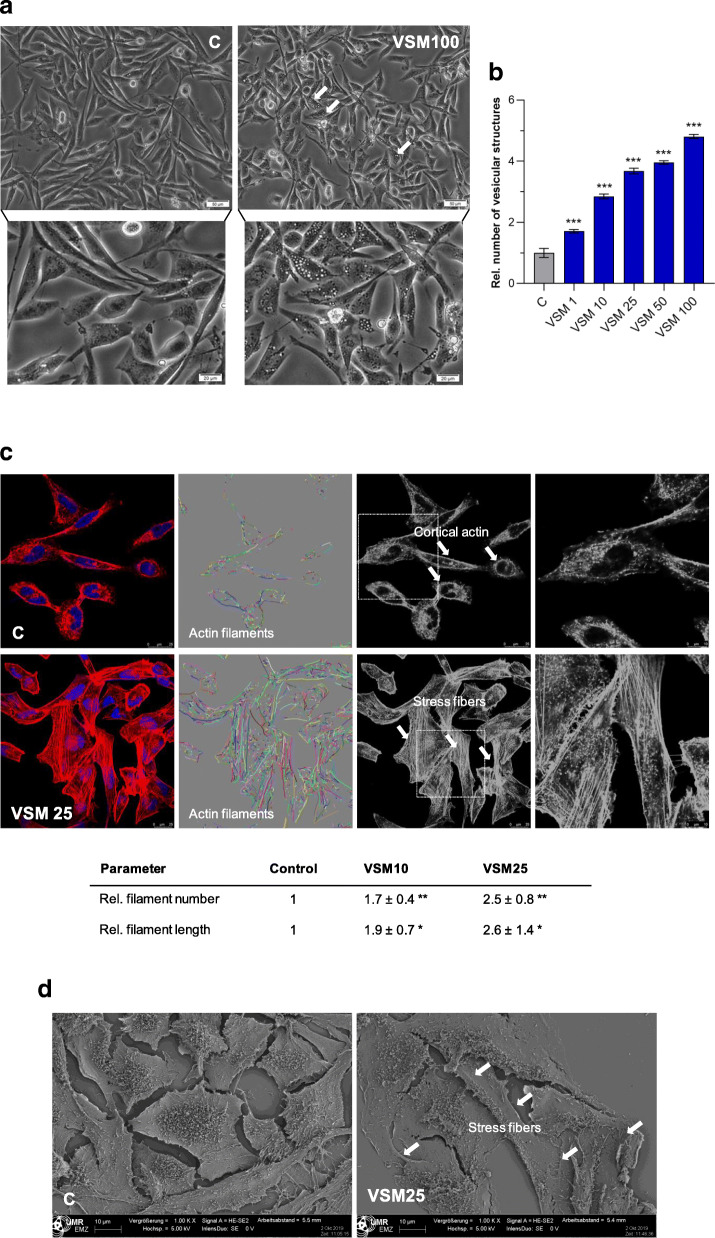
VSM treatment effects on RH-30 cell morphology. **a** Bright field images of VSM concentration series (1, 10, 25, 50 and 100 μg/ml VSM) treated RH-30 cells after 48 h compared to the solvent control (0.1% DMSO). **b** The number of vesicular structures in course of VSM treatment were relatively quantified of five bright field microscopical individual images. Mean ± SD, *n* = 5, *** *P* < 0.001, ** *P* < 0.01, * *P* < 0.05, significantly different compared to vehicle control, unpaired t-test. **c** Alterations of the cytoskeleton of RH-30 cells after 25 μg/ml VSM treatment for 48 h compared to the DMSO (0.1%) treatment control. F-actin organization was analyzed via confocal microscopy and was also mathematical processed with the software FilaQuant (University of Rostock, Institute of Mathematics, Mathematical Optimization, Rostock, Germany). F-actin fibers were labelled with Phalloidin Alexa 546 (red) and the cell nuclei with Hoechst (blue). F-actin filaments in the processed images are shown as colored lines. Mean ± SD, unpaired t-test, *n* = 3, ** *P* < 0.01, * *P* < 0.05. **d** Determination of morphological alterations on RH-30 cells with scanning electron microscopy after 48 h treatment with 25 μg/ml VSM extract compared to the vehicle control (0.1% DMSO)

The initial investigations revealed strong anti-migratory and anti-invasive effects for VSM (Fig. 2). Therefore, VSM was selected for studies focusing on cell morphology, cytoskeleton, and cellular compartments (Fig. 3). First, bright field microscopical analysis under VSM treatment revealed a significant concentration-dependent increasement of vesicular structures in the inner cell area around the nucleus (Figs. 3a – b, Additional file 1: Figure S1A ). RMA cells are known for its high metastatic potential and therefore in confocal microscopy untreated RH-30 cells displayed a motile and invasive cytoskeletal shape, which is demonstrated in the well-defined cortical actin in Fig. 3c. In contrast confocal as well as scanning electron microscopical imaging revealed a strong induction of stress fiber formation in RH-30 cells under 48 h VSM25 exposure (Fig. 3c and d), which was also verified via the mathematical quantification of the relative actin filament number and length with the software ‘FilaQuant’ (Fig. 3c). The quantification revealed significant actin-stabilizing properties of VSM10 and VSM25 shown by a significant 1.7- to 2.6-fold increase respectively in filament length and filament number. Furthermore, a stabilization of the actin cytoskeleton under Geni25 and Jaspl.10 (positive control) treated RH-30 cells was also examined by confocal microscopy (Additional file 1: Figure S1B).

### Influence of VSM treatment on the expression of selected biomarker

**Fig. 4:**
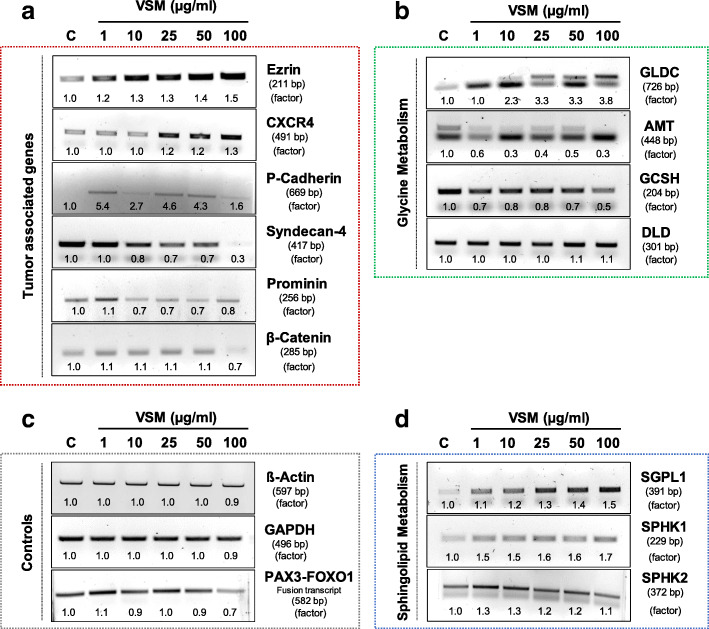
Analysis of concentration dependent effects of VSM on the transcript level in RH-30 cells. The expression factors were all determined densitometrically and normalized to the solvent control, which was set to 1. Transcript expression analysis of RH-30 cells after 48 h VSM treatment (1, 10, 25, 50 and 100 μg/ml) by RT-PCR of ß-actin and GAPDH (loading control); pax3-foxo1 (RMA marker); GLDC, AMT, GCSH and DLD (components of the glycine cleavage system); SGPL1, SPHK1 and SPHK2 (sphingolipid metabolism); as well as ezrin, CXCR4, P-cadherin, syndecan-4, prominin and β-catenin (metastasis and tumor signaling marker). (Representative images of three independent experiments, *n* = 3)

To get an insight in the underlying mode of action, different VSM concentrations (1, 10, 25, 50 and 100 μg/ml) were applied to RH-30 cells and its effects on the transcript as well as protein expression levels of selected molecular, metabolic, metastasis and tumor signaling markers were evaluated via RT-PCR (Fig. 4) and western blotting (Additional file 2: Figure S2). Transcript analysis revealed a linear, concentration-dependent increase of the glycine decarboxylase (GLDC), sphingosine-1-phosphate lyase (SGPL1), sphingosine-1-phosphate kinase 1 (SPHK1), ezrin and chemokine receptor type 4 (CXCR4), and in contrast a linear decrease of the aminomethyl transferase (AMT), P-cadherin and syndecan-4 transcripts (Fig. 4). The transcript level of the tumor stem cell marker prominin (CD133) was generally negatively affected by an expression decrease with an average of 20–30% at VSM concentrations in a range of 10–100 μg/ml. Treatment with the highest VSM concentration of 100 μg/ml revealed a strong decrease of the glycine cleavage system protein H (GCSH) (− 50%), ß-catenin (− 30%) and interestingly of the pax3-foxo1-fusion (− 30%) transcript expression level. The transcript levels of dihydrolipoyl dehydrogenase (DLD) and sphingosine-1-phosphate kinase 2 (SPHK2) as well as of GAPDH and ß-Actin loading, and housekeeping controls were not altered. Protein expression analysis revealed a decrease in the proliferation rate of RH-30 cells at VSM concentrations ≥10 μg/ml, which was occupied by a linear about 50–90% reduced Proliferating Cell Nuclear Antigen (PCNA) (Additional file 2: Figure S2C) and of 20–40% reduced stathmin (regulator of the cell cycle and cytoskeleton; oncogene) protein expression (Additional file 2: Figure S2A). As internal loading controls ß-actin labeling (Additional file 2: Figure S2C) and stainfree imaging technique (Additional file 2: Figure S2E) were used to ensure uniformly applied protein quantities.

### Metabolic alterations under VSM exposure

**Fig. 5:**
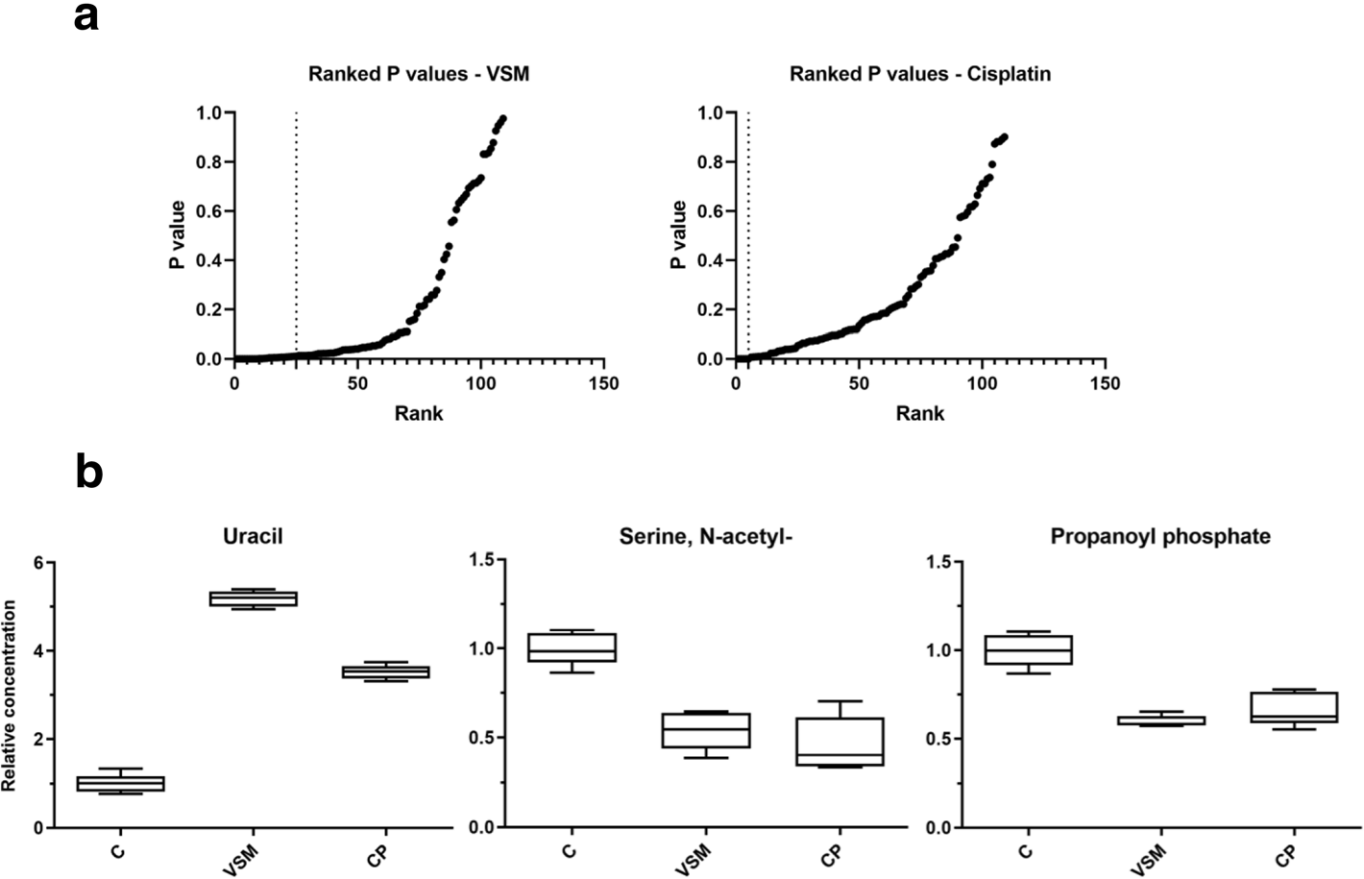
Metabolic profiling of VSM and cisplatin treated RH-30 cells. **a** *P*-value ranking according to the original FDR method of Benjamini and Hochberg (Q = 5%), **b** as well as the boxplot representations for 3 significantly different metabolites in RH-30 cells in course of 48 h VSM treatment (25 μg/ml) and cisplatin (CP) treatment (3.3 μM), relatively expressed to the median of the untreated controls (metabolite annotations are putative). The metabolic profiles of treated RH-30 cells show a similar responding in three metabolites to the VSM and cisplatin treatment. While uracil is 3.5 to 5-fold increased, N-acetyl serine and propanoyl phosphate are moderately decreased by 40–50% under cisplatin and VSM treatment

RH-30 cell metabolite levels after 48 h treatment with 25 μg/ml VSM or with 3.3 μM cisplatin were compared to untreated control cells (Fig. 5). Cisplatin is an alkylating and cytotoxic agent which is commonly used as chemotherapeutic drug in intermediate and high-risk RMS tumor treatment [1, 2]. For the analysis of VSM and cisplatin (CP) impact on the metabolic profile of RH-30 cells, a non-targeted investigation of intracellular metabolite pools of small polar compounds after methanolic extraction using Gas-Chromatography Atmospheric-Pressure-Chemical-Ionization Mass-Spectrometry (GC-APCI-MS) was conducted. In total, 109 different compounds were analyzed and 80 of them were identified by comparison against a spectra library of reference compounds. An unsupervised principal component analysis (PCA) on the raw metabolite data matrix was performed and revealed systematic differences between the metabolic profiles. The individual metabolic differences between CP and VSM treated cells were rather moderate. Therefore, each metabolite was further compared between the treatment and the control group and an ANOVA analysis followed by a multiple testing correction according to the FDR method of Benjamini-Hochberg (Q = 5%) was conducted (*p*-value ranking in Fig. 5a). The analysis revealed 25 significantly changed metabolites in VSM and only 5 significantly changed metabolites in cisplatin treatment (at *P* ≤ 0.01) compared to the non-treated RH-30 cell control (Fig. 5b and Additional file 3: Figure S3). The two treatments induced a similarly altered metabolite profile in the RH-30 cells compared to the untreated control cells. However, due to the increased scattering of the CP treatment values, probably due to technical artefacts, fewer metabolites were significantly altered compared to the VSM treatment. Three identified metabolites showing the strongest changes under VSM as well as under CP treatment, were annotated as uracil, N-acetyl serine, and propanoyl phosphate, thus provoked an analogous response to the cell metabolites (Fig. 5b).

### VSM treatment effects on healthy cells

**Fig. 6:**
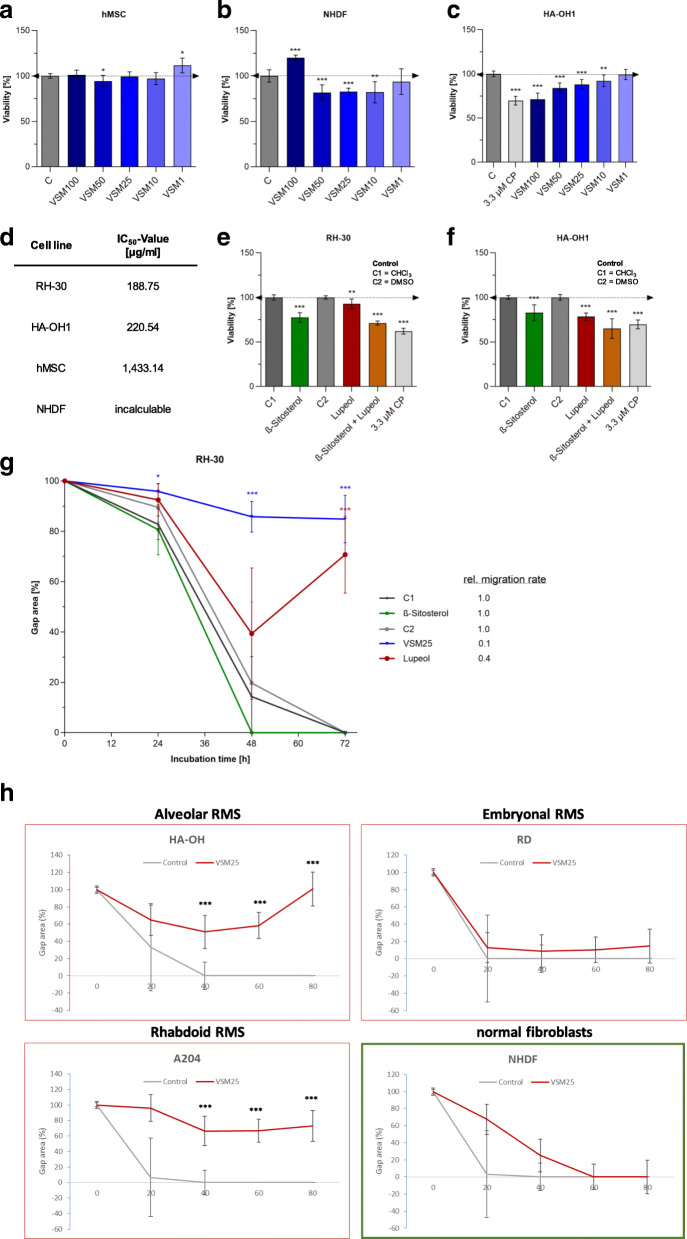
Migratory evaluation of VSM treatment on healthy control cells and three further RMS cell lines, and analysis of the pure VSM compounds ß-sitosterol and lupeol on the viability. Cell viability measurement via MTS-assay after treatment with VSM extract (1, 10, 25, 50 and 100 μg/ml) of the primary non-tumorigenic control cells: **a** human mesenchymal stem cells (hMSCs) and **b** human fibroblasts (NHDF), as well as of the RMA cell line: **c** HA-OH1. **d** IC_50_-value calculations for VSM on 48 h extract treated RMA cell line RH-30 (see Fig. 1a) and HA-OH1, as well as healthy hMSCs and NHDF control cells. Cell viability measurement via MTS-assay after 48 h treatment with isolated pure VSM compounds ß-sitosterol and lupeol (50 μg/ml) of the RMA cell line: **e** RH-30, and **f** HA-OH1. The solvent (0.1% DMSO for lupeol and 0.1% CHCl_3_ for ß-sitosterol) was used as negative control and set to 100%. Mean ± SD, *n* = 6–8, *** *P* < 0.001, ** *P* < 0.01, * *P* < 0.05, significantly different compared to vehicle control, unpaired t-test. **g** Evaluation of cell migration capacity and relative migration speed (ratio) of RH-30 cells during treatment with VSM extract (10 and 25 μg/ml) and pure VSM compounds (50 μg/ml ß-sitosterol and lupeol) for 3 days compared to the vehicle control (0.1% DMSO for VSM10, VSM25 and lupeol; 0.1% CHCl_3_ for ß-sitosterol). Mean ± SD, *n* = 6–8, *** *P* < 0.001, ** *P* < 0.01, * *P* < 0.05, significantly different compared to the control, unpaired t-test. **h** Migration assay of one another alveolar (HA-OH), embryonal (RD) and rhabdoid (A204) cell line in comparison to normal, primary fibroblasts (NHDF) under VSM25 treatment. Mean ± SD, *n* = 4–6, *** *P* < 0.001, ** *P* < 0.01, * *P* < 0.05, significantly different compared to the control, unpaired t-test

To test the effects of VSM on healthy cells, a concentration series of VSM (100, 50, 25, 10,1 μg/ml) was applied to human mesenchymal stem cells (hMSC) as well as human fibroblasts (NHDF) functioning as primary control lines (Fig. 6a-b). The hMSCs were not affected by the VSM treatment (Fig. 6a), whereas the cell viability of NHDF control cells were slightly influenced in a biphasic manner (Fig. 6b). Additionally, the VSM extract was incubated on a further RMA cell line HA-OH1, to verify the results got by RH-30. VSM extract treatment of HA-OH1 RMA cells induced a significant decreased viability rate in a range of 10–30%, analogue to the results of the RH-30 cells in Fig. 1a (Fig. 6c). Further, the IC50-values in course of VSM concentration series were calculated by dose-response curves (Additional file 4: Figure S4), which resulted in slightly pharmacological potent (> 100 μg/ml) IC50-values for the RMA cell lines RH-30 (188.75 μg/ml) and HA-OH1 (220.54 μg/ml) as well as non-tumorigenic primary cells (hMSC: 1433.14 μg/ml) (Fig. 6d).

### Analysis of pure VSM compounds

Furthermore, the treatment effects of the two main VSM components: ß-sitosterol and lupeol were tested exemplary on the two RMA cell lines RH-30 and HA-OH1. Therefore, the metabolic activity as indirect sign for cell viability after 48 h treatment with 50 μg/ml single substance was evaluated first (Fig. 6e and f). Afterwards, the single substance effects compared to VSM extract effects on the cell migration capacity of the RH-30 cells were tested (Fig. 6g). Treatment with ß-sitosterol induced a significant viability decrease of 20% in both RMA cell lines (Fig. 6e and f). The lupeol treatment induced a significant viability decrease of 10% in RH-30 (Fig. 6e) and 20% in HA-OH1 (Fig. 6f). Moreover, the combinatorial treatment induced a significant decreased viability rate of 30% in RH-30 and 35% in HA-OH1 cells (Fig. 6e and f). Only the treatment with 50 μg/ml lupeol revealed a significant anti-migratory effect on RH-30 cells (Fig. 6g).

### VSM anti-migratory effects on further RMS cell lines

In addition to the established alveolar rhabdomyosarcoma cell line RH30 one another alveolar (HA-OH), embryonal (RD) and rhabdoid (A204) cell line in comparison to normal, primary fibroblasts (NHDF) were used to study anti-migratory effects of VSM25 (Fig. 6h). HA-OH and A204 cell migration was impaired to the same extent as is evident for Rh30 (Fig. 6g). Cells start to migrate into the gap with the first 2 h and thereafter migration was stopped, and cells start to detach after 3 days in assay medium containing VSM25. In contrast, the embryonal cell line RD was not affected by VSM25. RD cell migration speed was comparable to the control treatment. For the primary fibroblasts, a migration reduction was detectable within the first 24 h, thereafter the gap closed like the control did. Notably, during the migration assay fibroblasts were cultivated in specific fibroblast medium and not in the charcoal treated medium used for the cancer cell lines. Interestingly, VSM25 has no significant influence on the initial adhesion capacity of the cells (SFig. 5).

## Discussion

The tendency of RMA cells to a rapid and severe metastasis formation is one of the major therapy challenges in RMA treatment. Possible therapy strategies are limited and chemoresistance due to genetical and metabolic alterations are frequently leading to tumor recurrences and metastatic invasion. In conclusion, a great demand for novel agents which can prevent metastasis is apparent [38]. Based on our previous anti-cancer studies in breast and bone cancer, we performed an anti-tumor screening with the focus on the anti-metastatic properties of two selected medicinal plant extracts: *Vincetoxicum arnottianum* Wight and *Linum usitatissimum* and four plant-derived compounds: genistein, daidzein, matairesinol and secoisolariciresinol on the pediatric RMA cell line RH-30 [21–23]. The initial screening revealed the VSM extract as the most potential anti-metastatic agent illustrated by the significant reduction of the migration, invasion, and colony formation in RH-30 cells (Fig. 2). After VSM treatment RMA cells were stabilized proven by a significant enhanced actin stress fiber formation (Fig. 3c and d). Moreover, with increasing VSM concentrations (1–100 μg/ml) an accumulation of vesicular structures could be found circularly distributed around the nucleus of RH-30 cells (Fig. 3a and b, as well as Additional file 1: Figure S1A). This might be due to the stimulation of the lysosomal network, resulting in an increased aggregation or activity induction of lysosomes. This phenomenon was previously reported in our study of anti-migratory effects on breast and bone cancer [21]. Notably, VSM exposure revealed low cytotoxicity in heathy breast and bone cells, as well as human mesenchymal stem cells (Fig. 6a). Since VSM also harbor strong anti-metastatic properties in RMA cells (Figs. 1, 2 and 3), VSM treatment bears anti-cancer potential for a broad spectrum of different cancer types. The anti-carcinogenic effect ascribed to VSM (*Vincetoxicum arnottianum* Wight) or closely related plants of the plant family Apocynaceae has its origin in traditional healing medicine, where it is also used for a wide variety of wounds and diseases like malaria, diabetes, diarrhea, etc. [21, 39]. The main compounds in the methanolic whole plant extract of VSM were recently identified as β-Sitosterol and Lupeol [29]. Both compounds were further tested for its effects on cell viability and cell migration of RMA cells (Figs. 6e-g). However, the analysis revealed that these two substances do not exert their full anti-tumor properties on RMA cells (Figs. 6e-g). Only in the natural context, i.e., applying the multi-substance mixture of the VSM extract, these migrastatic effects can be achieved. These results offer a research area for insightful investigation to understand the chemical constituents present in the VSM that exhibit such activity.

The molecular mechanism of VSM was determined on transcript and protein expression level, by analyzing selected tumor biomarkers (Fig. 4 and Additional file 2: Figure S2) and metabolite levels (Fig. 5). High VSM concentrations ≥50 μg/ml slightly decreased the pax3-foxo1 fusion transcript expression (Fig. 4c), which can be due to direct or indirect VSM effects on upstream activator/ kinase phosphorylation activity [40]. A reduced pax3-foxo1 expression demonstrably leads to induction of myogenic differentiation, growth arrest and as consequence to apoptosis [41, 42]. Furthermore, a decreased proliferation of RH-30 cells after VSM exposition was evident by linear reduced PCNA protein expression (Additional file 2: Figure S2C) as well as reduced capability of C1-body generation for proliferation purposes through glycine decarboxylation via the glycine cleavage system (GCS) components GLDC, AMT, GCSH and DLD (Fig. 4b and Additional file 2: Figure S2B). Cancer cells exhibit a preference for increased glycine consumption to satisfy their high demand for C1 bodies to ensure a high capacity for proliferation and continuous growth [34, 43, 44]. Further, this study demonstrated that the transcript and protein expression level of the sphingosine-1-phosphate (S1P) metabolizing enzyme sphingosine-1-phosphate lyase (SGPL1) was significantly increased in a concentration dependent manner (Fig. 4d and Additional file 2: Figure S2D). S1P is a bioactive sphingolipid and second messenger which is involved in cellular signaling and regulation processes of cell motility, angiogenesis, proliferation, growth, cytoskeletal organization, as well as adhesion-dependent cell survival [45–48]. Moreover, high concentrations of S1P or deficiencies in S1P degradation by SGPL1 have been associated with cancer cell progression, directed chemoattraction and promotion of chemo-resistance mechanism [31, 48–50]. S1P is also discussed as potential chemoattractant to force metastatic invasion of RMS cells [31, 51]. In this context, an increased SGPL1 expression under VSM treatment positively correlates with the reduced capacity of RH-30 cell migration, invasion, and colony formation. This is due to the reduced ability for S1P-directed chemoattraction induced by the irreversible SGPL1-mediated S1P cleavage to the non-sphingolipid molecules hexadecenal and ethanolamine phosphate. Further observations underlined that VSM exposure decreases the metastatic behavior of RH-30 cells by reducing the transcript level of syndecan-4 and prominin and the protein expression level of CXCR4 and stathmin (Fig. 4a and Additional file 2: Figure S2A). The chemokine receptor CXCR4 and prominin (CD133) expression has been found to affect the metastatic potential of RMA cells, whereas syndecan-4 and stathmin expression can predict the clinical outcome of many cancer types [52–55]. Finally, a metabolic profiling and comparative consideration of VSM and standard treatment with cisplatin compared to non-treated cells confirmed that VSM as well as cisplatin treatment only moderately affects primary metabolism of the RH30 cells (Fig. 5). Notably, cisplatin is used in RMS regimes in some clinics but will be in future not the most promising chemotherapeutic drug. However, in both treatments the three metabolites uracil, N-acetyl serine and propanoyl phosphate were affected (Fig. 5b). The uracil levels were increased and may indicate an impaired pyrimidine homeostasis which can foster DNA strand breaks due to incorrect substitution and negative cytotoxic effects [56, 57]. The decreased levels of N-acetyl serine could be an indication for protein degradation. Both metabolite alterations were also found in our previous study concerned on RH-30 cell treatment with the methanolic root extract of *Berberis orthobotrys* (BORM), a plant derived extract with anti-cancer properties [30]. The similar metabolite profiles under VSM, cisplatin and BORM treatment could be an indication of a positive response to therapy. Taken together, the results of this study indicate that VSM treatment stabilizes the actin cytoskeleton whereby the motility, invasion, colony formation, growth and proliferation levels of RH-30 cells were reduced, which is followed by a metabolic shut down. However, this study is limited due to the fact that so far, we have only been able to identify the effectiveness of the VSM root extract and we have not yet been able to identify the effective individual substances. Therefore, chemical analysis is being promoted to elucidate the central VSM anti-cancer compound.

## Conclusions

Our results reveal the methanolic whole plant extract of *Vincetoxicum arnottianum* (VSM) as potent migrastatic drug candidate with actin filament stabilizing properties and low side effects for the treatment of pediatric alveolar rhabdomyosarcoma.

## Supplementary Information

**Additional file 1: Figure S1.** Morphological alterations under VSM, Geni and Jaspl. treatment. **(A)** Bright field images of VSM concentration series (1, 10, 25, 50 and 100 μg/ml VSM) treated RH-30 cells after 48 h compared to the solvent control (0.1% DMSO). **(B)** Fluorescence microscopical imaging of the actin cytoskeleton of RH-30 cells after treatment with 25 μM Geni and 10 nM Jaspl. for 48 h compared to the vehicle control (0.1% DMSO). F-actin fibers were labelled with Phalloidin Alexa 546 (red) and the cell nuclei with Hoechst (blue).

**Additional file 2: Figure S2.** Concentration dependent effects of VSM on RH-30 cell protein level. The expression factors were all determined densitometrically and normalized to the solvent control, which was set to 1. Determination of concentration depended effects of VSM treatment (1, 10, 25, 50 and 100 μg/ml) on **(A)** ezrin, CXCR4, P-cadherin, stathmin, **(B)** AMT, GCSH, **(C)** *β*-actin, PCNA, and **(D)** SGPL1 protein expression in 48 h extract treated RH-30 cells. (Representative images of three independent experiments, *n* = 3) **(E)** Stain-free image of polyacrylamide gel functions a loading control (10 μg protein per lane were applied to the protein gel).

**Additional file 3: Figure S3.** Further affected metabolites under VSM treatment. Additional boxplot representation for further 12 significantly different metabolites in RH-30 cells in course of 48 h VSM treatment (25 μg/ml), relatively expressed to the median of the untreated controls (metabolite annotations are putative).

**Additional file 4: Figure S4.** Original VSM dose-response curves of calculated IC_50_-values. 48 h extract treated RMA cell lines **(A)** RH-30 and **(B)** HA-OH1, as well as the primary non-tumorigenic control cells **(C)** human mesenchymal stem cells (hMSC) and **(D)** human fibroblasts (NHDF).

**Additional file 5: Figure S5.** Influence of VSM25 treatment on new adhesion capacity. Initial adhesion of Rh30, RD, HA-OH and A204 cells was analyzed under control, VSM25 pretreated, VSM25 post-treated and VSM25 permanent treated conditions. Mean ± SD, *n* = 4–5.

## Data Availability

The data sets supporting the conclusions of this article are presented in this main paper. And the supporting materials can be obtained upon request via email to the corresponding author.

## Abbreviations

AMT: Aminomethyl transferase
ANOVA: One-way analysis of variance
BORM: *Berberis orthobotrys*
CHCl_3_: Chloroform
CP: Cisplatin
CXCR4: Chemokine receptor type 4
Daid: Daidzein
DLD: Dihydrolipoyl dehydrogenase
DMSO: Dimethyl sulfoxide
DNA: Deoxyribonucleic acid
EtOH: Ethanol
FDR: False discovery rate
GC-APCI-MS: Gas-chromatography atmospheric-pressure-chemical-ionization mass-spectrometry
GC-MS: Chromatography–mass spectrometry
GCS: Glycine cleavage system
GCSH: Glycine cleavage system protein H
Geni: Genistein
GLDC: Glycine decarboxylase
hMSC: Human mesenchymal stem cells
IC50: Inhibitory concentration of 50% population
Jaspl.: Jaspamide
LC-MS: Liquid chromatography–mass spectrometry
LW: *Linum usitatissimum*
Mata: Matairesinol
MeOH: Methanol
MS: Mass-Spectrometry
MTS: 3-(4, 5-dimethylthiazol-2-yl)-5-(3-carboxymethoxyphenyl)-2-(4-sulfophenyl)-2H-tetrazolium
NMR: Nuclear magnetic resonance spectroscopy
Pax3/7-foxo1: Chimeric paired box 3/7–forkhead box O1 gene
PCA: Principal component analysis
PCNA: Proliferating cell nuclear antigen
RMA: Alveolar rhabdomyosarcoma
RMS: rhabdomyosarcoma
RNA: Ribonucleic acid
RT-PCR: Reverse transcription polymerase chain reaction
S1P: Sphingosine-1-phosphate
SD: Standard deviations
Seco: secoisolariciresinol
SEM: Scanning electron microscopy
SGPL1: Sphingosine-1-phosphate lyase
SPHK1: Sphingosine-1-phosphate kinase 1
SPHK2: Sphingosine-1-phosphate kinase 2
VSM: *Vincetoxicum arnottianum*
