# Supplementary figures and images for: Vincetoxicum arnottianum modulates motility features and metastatic marker expression in pediatric rhabdomyosarcoma by stabilizing the actin cytoskeleton
Source: BMC Complement Med Ther. 2021 May 4;21:136. doi: 10.1186/s12906-021-03299-x (PMC8097906; doi:10.1186/s12906-021-03299-x)

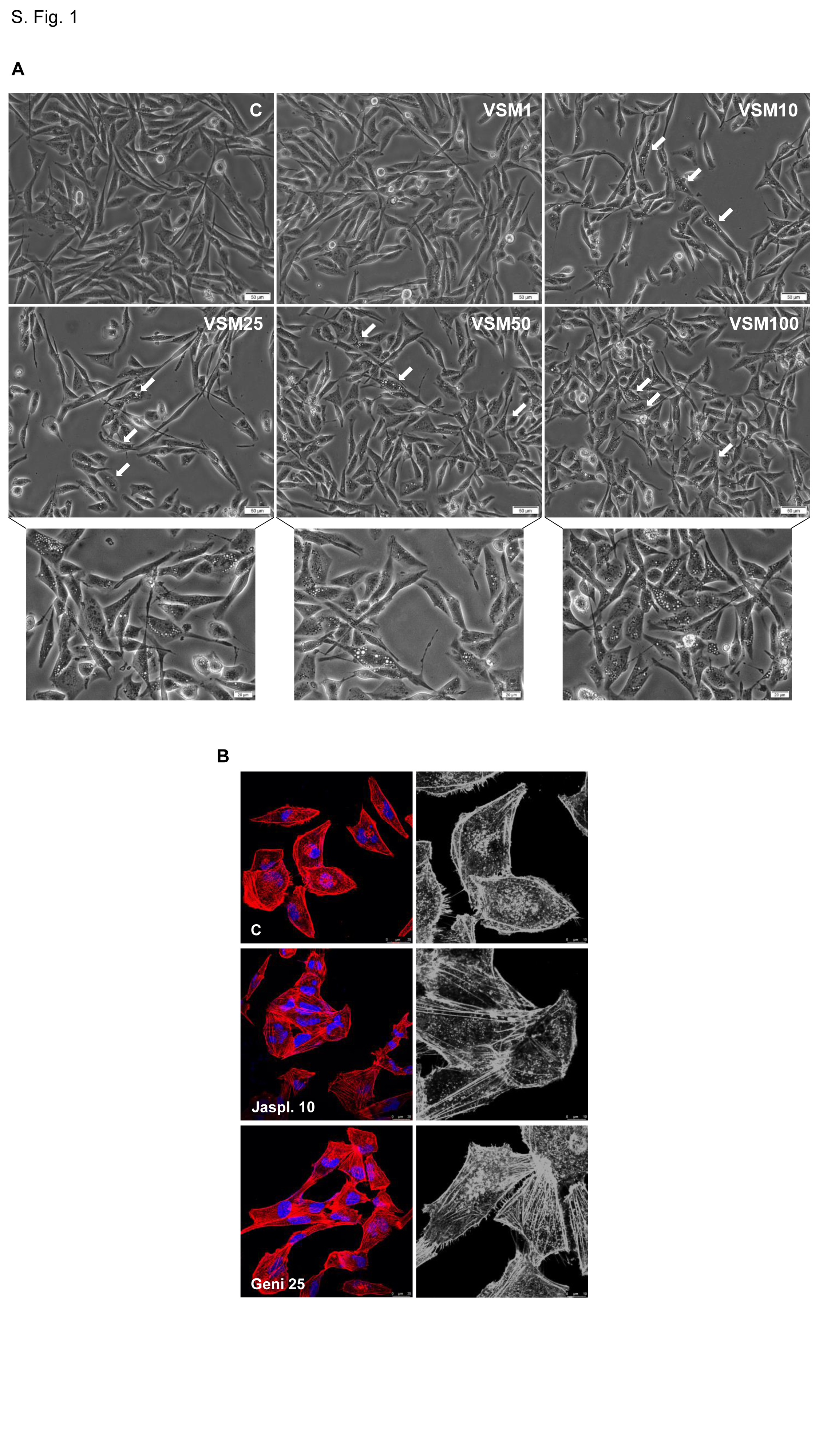

Supplement: Supplementary file 1 — Additional file 1: Figure S1. Morphological alterations under VSM, Geni and Jaspl. treatment. (A) Bright field images of VSM concentration series (1, 10, 25, 50 and 100 μg/ml VSM) treated RH-30 cells after 48 h compared to the solvent control (0.1% DMSO). (B) Fluorescence microscopical imaging of the actin cytoskeleton of RH-30 cells after treatment with 25 μM Geni and 10 nM Jaspl. for 48 h compared to the vehicle control (0.1% DMSO). F-actin fibers were labelled with Phalloidin Alexa 546 (red) and the cell nuclei with Hoechst (blue). [file 12906_2021_3299_MOESM1_ESM.tif]

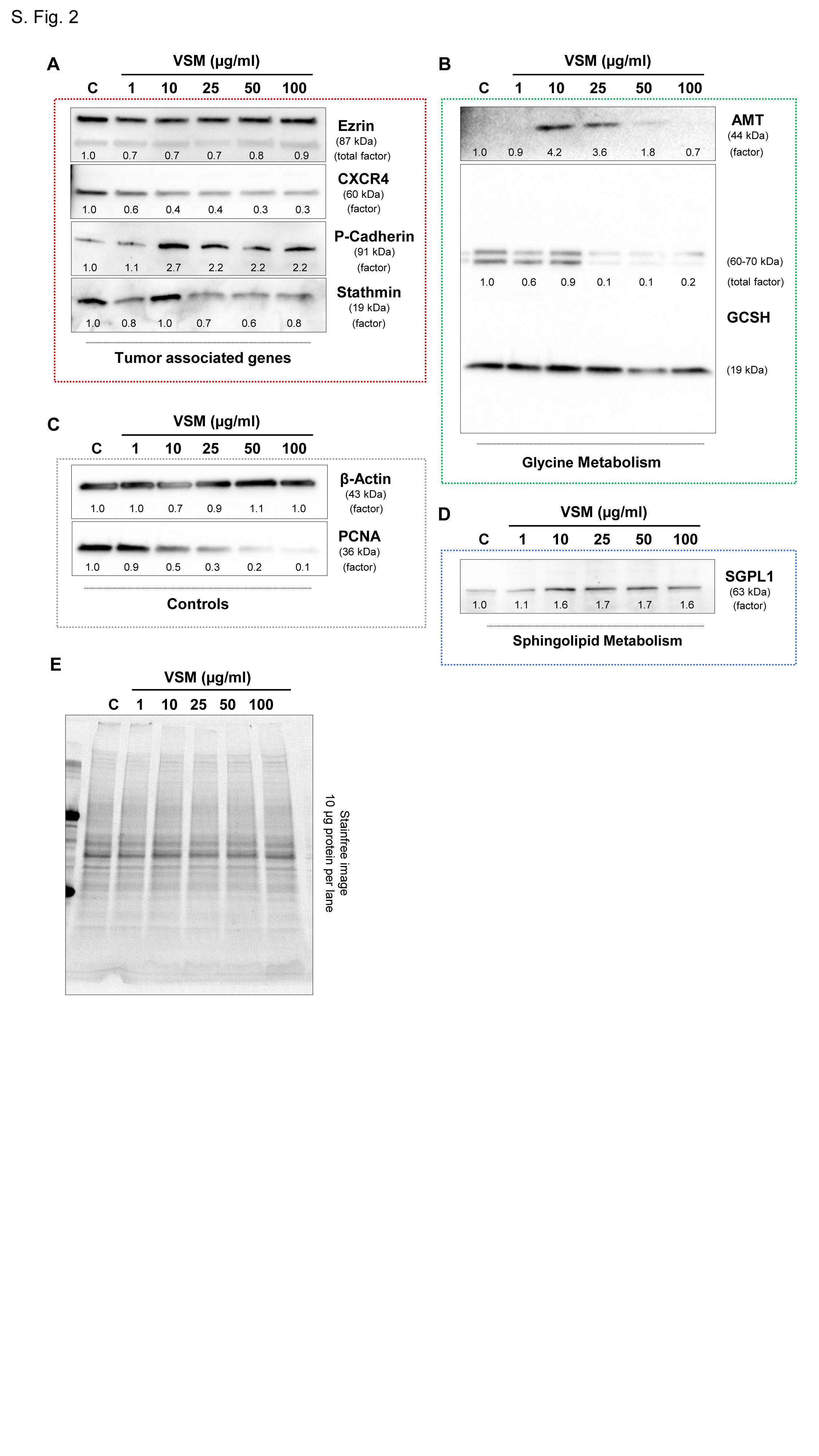

Supplement: Supplementary file 2 — Additional file 2: Figure S2. Concentration dependent effects of VSM on RH-30 cell protein level. The expression factors were all determined densitometrically and normalized to the solvent control, which was set to 1. Determination of concentration depended effects of VSM treatment (1, 10, 25, 50 and 100 μg/ml) on (A) ezrin, CXCR4, P-cadherin, stathmin, (B) AMT, GCSH, (C) β-actin, PCNA, and (D) SGPL1 protein expression in 48 h extract treated RH-30 cells. (Representative images of three independent experiments, n = 3) (E) Stain-free image of polyacrylamide gel functions a loading control (10 μg protein per lane were applied to the protein gel). [file 12906_2021_3299_MOESM2_ESM.tif]

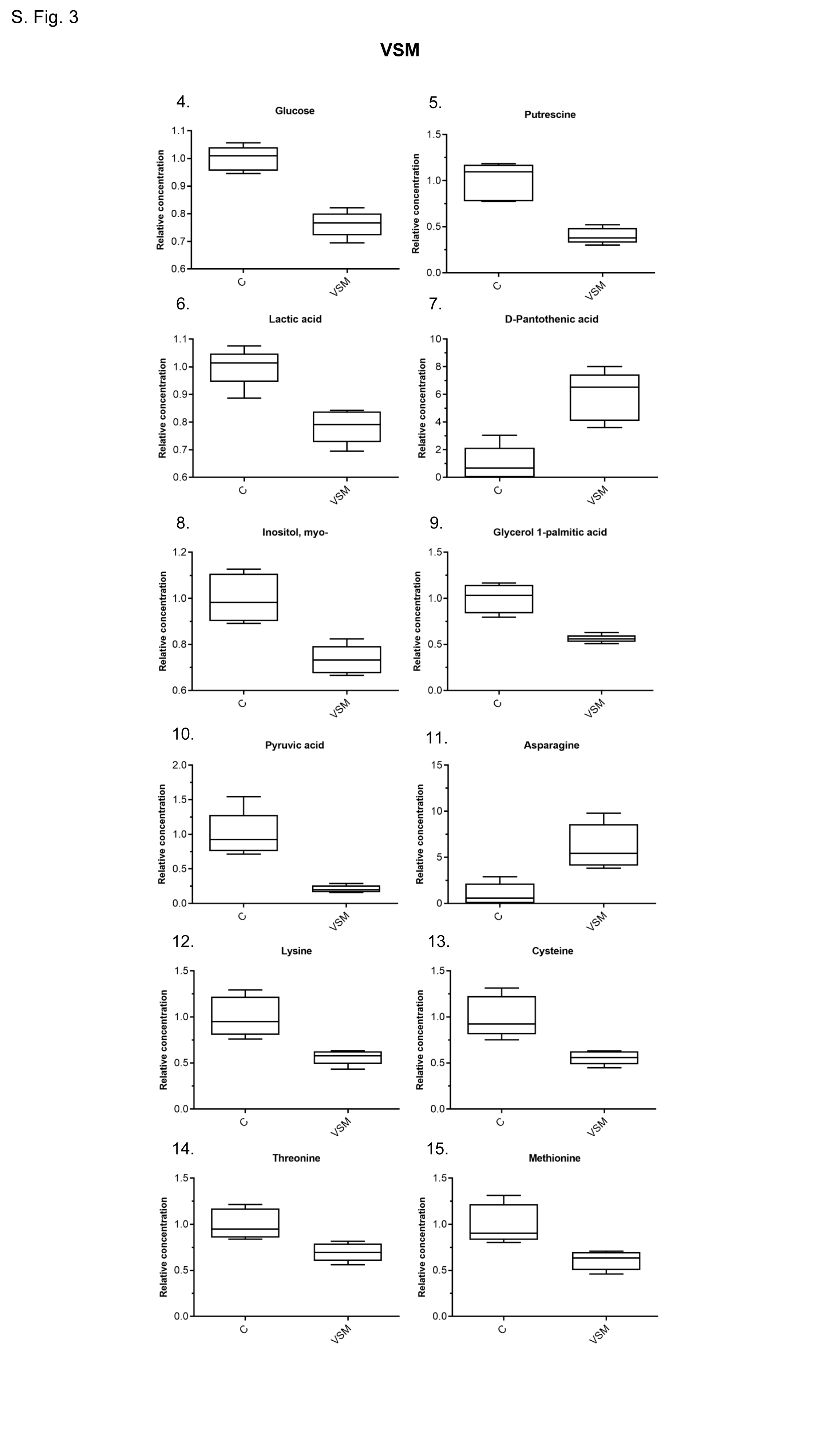

Supplement: Supplementary file 3 — Additional file 3: Figure S3. Further affected metabolites under VSM treatment. Additional boxplot representation for further 12 significantly different metabolites in RH-30 cells in course of 48 h VSM treatment (25 μg/ml), relatively expressed to the median of the untreated controls (metabolite annotations are putative). [file 12906_2021_3299_MOESM3_ESM.tif]

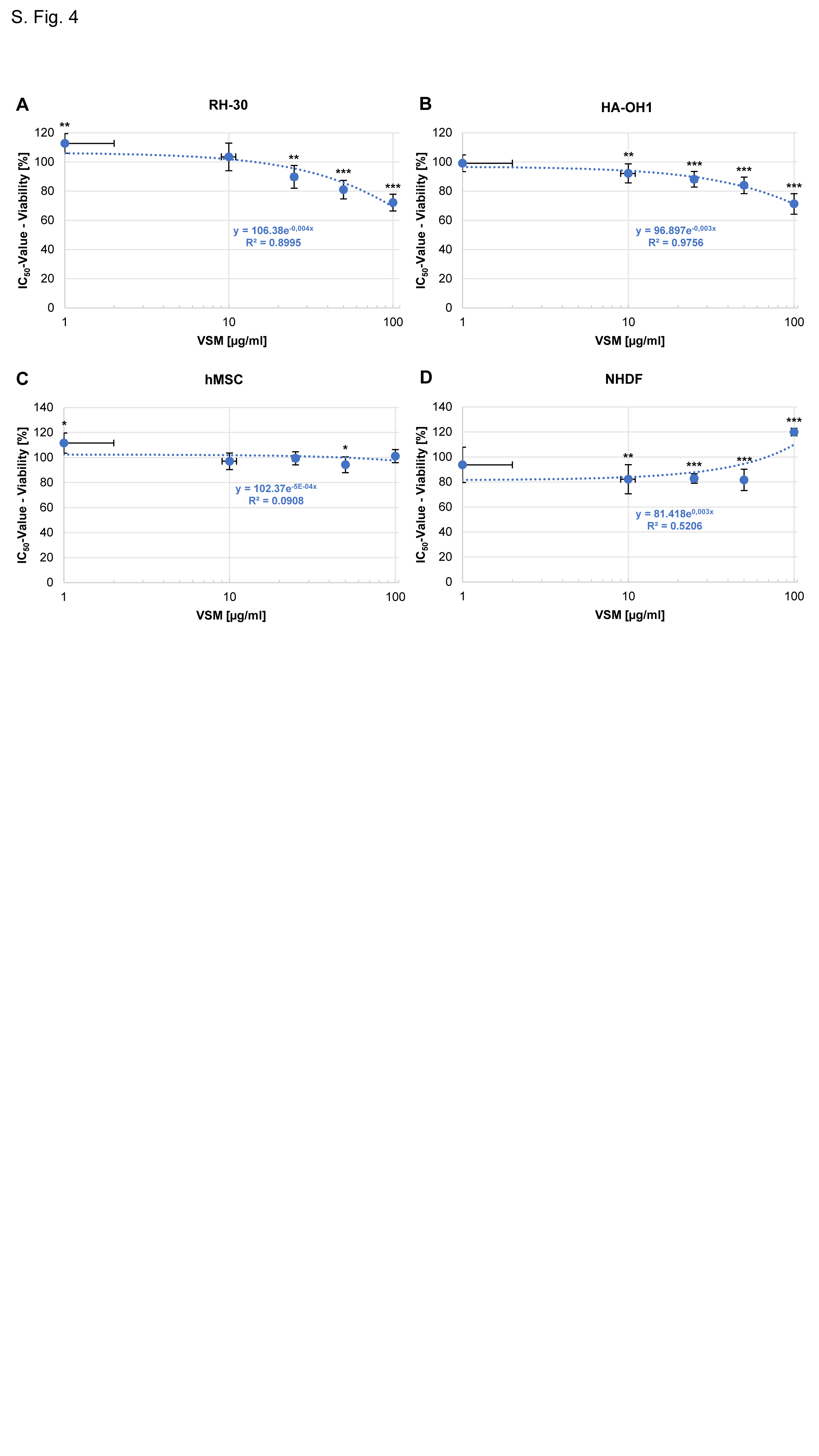

Supplement: Supplementary file 4 — Additional file 4: Figure S4. Original VSM dose-response curves of calculated IC50-values. 48 h extract treated RMA cell lines (A) RH-30 and (B) HA-OH1, as well as the primary non-tumorigenic control cells (C) human mesenchymal stem cells (hMSC) and (D) human fibroblasts (NHDF). [file 12906_2021_3299_MOESM4_ESM.tif]

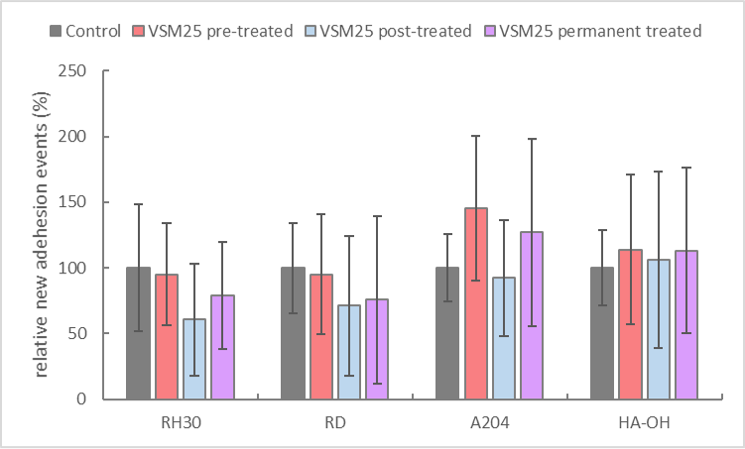

Supplement: Supplementary file 5 — Additional file 5: Figure S5. Influence of VSM25 treatment on new adhesion capacity. Initial adhesion of Rh30, RD, HA-OH and A204 cells was analyzed under control, VSM25 pretreated, VSM25 post-treated and VSM25 permanent treated conditions. Mean ± SD, n = 4–5. [file 12906_2021_3299_MOESM5_ESM.tif]
